# Novel 2-Pyrazoline Derivatives as Potential Antibacterial and Antifungal Agents

**DOI:** 10.4103/0250-474X.40344

**Published:** 2008

**Authors:** Suvarna Kini, A. M. Gandhi

**Affiliations:** Department of Pharmaceutical Chemistry, Manipal College of Pharmaceutical Sciences, Manipal - 576 104, India

**Keywords:** Pyrazolines, antibacterial agents, antifungal agents

## Abstract

The 1,3,5-trisubstituted-2-pyrazolines were synthesized by refluxing isoniazid with various substituted diarylchalcones in N,N-dimethylformamide at 120-140°. The physical and spectral data such as M.P., R_f_, elemental analysis, IR, NMR and Mass was obtained for the synthesized compounds and the structures were confirmed. The screening of the synthesized compounds for antimicrobial activity was performed against *Staphylococcus aureus, Bacillus subtilis, Pseudomonas aeruginosa, Escherichia coli* and *Aspergillus niger*.

2-pyrazolines are reported as antibacterial^1^, antifungal^2^,^3^,^4^, antimicrobial^5^, antiviral^6^, anti-arthritis^7^ and antiinflammatory^7^,^8^ agents. Encouraged by these facts, we selected to work on 1,3,5-trisubstituted-2-pyrazolines with different substitutions on the phenyl ring. The antibacterial activity and antifungal activity was screened by cup-plate agar diffusion method and the zone of inhibition for each microorganism at different concentrations of the compounds was measured.

Melting points of the compounds were determined on a Toshniwal Scientific melting point apparatus and are uncorrected. Purity of the compounds was checked by Thin Layer Chromatography using precoated Merck Silica gel GF_254_ micro TLC plates and spots were detected under UV. UV spectra were recorded on UV/Vis spectrophotometer (Shimadzu), UV-1601 PC, IR spectra were recorded in KBr disc on a FTIR_8300, KBr Press (Shimadzu) spectrophotometer at Manipal College Of Pharmaceutical Sciences, Manipal. ^1^H NMR spectra (DMSO-d_6_) were obtained using Varian 300 MHz Mercury NMR spectrometer from Shimadzu Analytical Technique Center, Department of Chemistry, Pune University, Pune. Mass spectra of the synthesized compounds were obtained using Mass Spectrometer, Shimadzu QP5050 from Shimadzu Analytical Technique Center, Department of Chemistry, Pune University, Pune. Chemicals were obtained from S.D. Fine Chemicals Ltd dealers from Mangalore.

Synthesis of 1,3,5-trisubstituted-2-pyrazolines [compound P13 (5-(4-fluorophenyl)-3-(4-fluorophenyl)-1-isonicotinoyl-pyrazoline)]^9^,^10^ was performed by taking equimolar quantities (0.02 mol) of 4-fluoro benzaldehyde and 4-fluoro acetophenone in a 250 ml conical flask and dissolving in minimum amount of 95% ethanol. The mixture was then cooled and 8 ml of 10% potassium hydroxide solution was added. The mixture was stirred over a magnetic stirrer for a period of 24 h and left in an ice chest overnight. On the next day, the mixture was poured into a beaker containing ice-cold water. Then the aqueous layer was acidified with conc. HCl. The precipitated chalcone (1-(4-fluorophenyl)-3-(4-fluorophenyl)-2-propen-1-one) was filtered at pump and recrystallized from aqueous methanol. IR (KBr)cm^−1^:1598.9 (C = C), 1660.6 (C = O), 3072.4 (C-H). (1-(4-fluorophenyl)-3-(4-fluorophenyl)-2-propen-1-one) (0.05 mol) was added to isoniazid (0.1 mol) in a 100 ml R.B.F. containing 30 ml of N, N-dimethylformamide. The mixture was refluxed at 120° to 140° for a period of 8-10 h. After cooling, the reaction mixture was poured into a beaker containing ice-cold water and the product separated was scooped off and dried over a clean filter paper. The pyrazoline obtained was recrystallized using benzene-petroleum ether. Single spot on TLC established the purity of the compound. Solvent system used was Hexane: ethyl acetate in 4:1 ratio. IR (KBr)cm^−1^: 1641.3 (N-C = O), 1595.0 (C = N), 1215.1 (C-N), 2914.2 (C-H,CH_2_),^1^H NMR (δ values) ppm: aromatic hydrogens 6.7-8.6 δ (m, 12H,Ar-H), CH_2_ methylene 3.4 δ (s, 2H, CH_2_), CH methine 5.6 δ (d, 1H,CH-Ar). The other derivatives were prepared in a similar way with different substituted chalcones according to the scheme of synthesis given in fig. 1. The physical data of the synthesized compounds is given in Table 1.

**Fig. 1:**
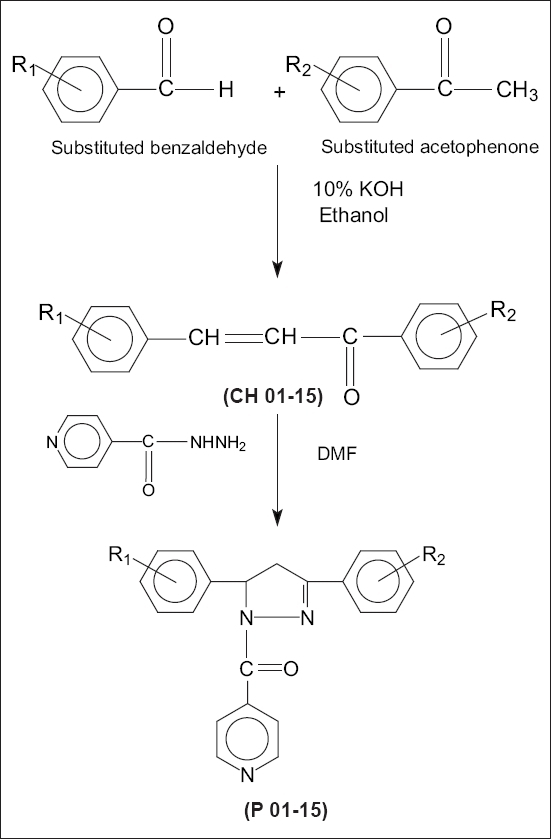
Scheme for pyrazoline synthesis. Where, R_1_ = -H, -Cl(m), -Cl(p), -F(p), -OCH_3_(p), -OCH_3_(m, p), - N(CH_3_)_2_(p), -NO_2_(m) R_2_ = -F(p), -Cl(p), -Br(p), -OCH_3_(p).

**TABLE 1:** PHYSICAL DATA OF THE SYNTHESIZED PYRAZOLINES

Pyrazoline	R_1_	R_2_	R_f_ Value	m.p. (°)	% Yield
P01	-F (p)	-Cl (p)	0.73	132-133	51
P02	-Cl (m)	-Cl (p)	0.64	123-125	55
P03	-OCH_3_ (p)	-Cl (p)	0.77	172-174	58
P04	-N(CH_3_)_2_ (p)	-Cl (p)	0.11	163-165	49
P05	-Cl (p)	-Cl (p)	0.17	86-88	34
P06	-OCH_3_ (m,p)	-Cl (p)	0.11	166-167	50
P07	-N(CH_3_)_2_ (p)	-OCH_3_ (p)	0.17	188-190	52
P08	-Cl (p)	-OCH_3_ (p)	0.16	214-215	59
P09	-NO_2_ (m)	-OCH_3_ (p)	0.31	170-172	36
P10	-OCH_3_ (m,p)	-OCH3 (p)	0.30	188-189	47
P11	-F (p)	-Br (p)	0.15	111-113	53
P12	-N(CH_3_)_2_ (p)	-Br (p)	0.14	96-98	32
P13	-F (p)	-F (p)	0.19	112-114	44
P14	-Cl (p)	-F (p)	0.15	162-163	65
P15	-OCH_3_ (p)	-F (p)	0.12	157-158	58

All the compounds gave satisfactory spectral and elemental data

All the synthesized compounds were screened *in vitro* for antibacterial activity against *Staphylococcus aureus, Bacillus subtilis, Pseudomonas aeruginosa*, *Escherichia coli* at the concentrations 200, 300, 400 and 500 mg/ml and for antifungal activity against *Aspergillus niger* at 100, 200, 300, 400 μg/ml by cup-plate agar diffusion method^11^. The concentrations used in screening were chosen after determining the MICs of each compound. The solvent used was dimethylsulfoxide (DMSO) further diluted with water. Muller Hinton agar was used as the growth medium for the bacterial species and Sabouraud's agar was the growth medium for the fungal species. DMSO was used as a control for all the type of microorganisms. The control showed no activity against the strains of microorganisms used. Antimicrobial activity and antifungal activity was measured as a function of diameter of zone of inhibition (mm). The results were compared with standard drugs ciprofloxacin for antibacterial activity and fluconazole for antifungal activity by measuring the zone of inhibition in mm at 200 and 100 μg/ml respectively. At 200 μg/ml, compounds P14 and P15 were found most effective of the synthesized compounds against *S. aureus*, (zone of inhibition 15 mm) and compound P14 is the most effective among the synthesized compounds at 200 μg/ml against *B. subtilis*, (zone of inhibition 16 mm). Compounds P02, P08, P09, P10, P11 were the most effective among the synthesized compounds against *E. coli*, (zone of inhibition 15 mm) and compound P05 was the most effective at 200 μg/ml among the synthesized compounds against *P. aeruginosa*, (zone of inhibition 21 mm). At 100 μg/ml the maximum diameter of zone of inhibition (20 mm) against *Aspergillus niger* was observed for compound P08; indicating that, it is the most effective among the synthesized compounds against *Aspergillus niger*. The results of the antibacterial and antifungal activity are given in Table 2.

**TABLE 2:** RESULTS OF *IN VITRO* ANTIBACTERIAL AND ANTIFUNGAL ACTIVITY

Code	*S. aureus*				*B. subtilis*				*E. coli*				*P. aeruginosa*				*A. niger*			
					
	B	C	D	E	B	C	D	E	B	C	D	E	B	C	D	E	A	B	C	D
P01	12	13	15	16	13	14	16	17	12	13	14	16	-	-	-	-	12	13	14	16
P02	14	16	18	22	13	14	15	17	15	17	19	21	15	16	18	20	-	-	-	-
P03	13	14	15	17	14	16	17	19	12	14	16	17	-	-	-	-	12	13	15	16
P04	13	14	15	17	12	13	15	16	12	13	14	16	18	20	22	26	14	15	16	18
P05	12	14	16	17	-	-	16	19	13	14	16	17	21	24	26	28	-	-	-	-
P06	12	13	14	16	12	13	14	15	12	13	14	15	20	22	23	25	-	-	-	-
P07	-	-	-	13	12	13	14	16	12	13	14	16	15	19	22	25	19	21	23	24
P08	12	13	15	16	12	14	15	17	15	18	21	23	-	-	-	-	20	22	24	26
P09	12	14	16	18	-	-	-	-	15	16	17	19	12	16	20	23	16	18	20	22
P10	13	15	17	20	-	12	14	16	15	17	19	21	18	21	24	26	15	17	19	20
P11	12	13	14	16	12	13	15	16	15	17	21	23	17	19	22	24	15	16	18	19
P12	12	14	15	17	-	-	12	14	12	14	15	17	17	20	23	25	16	18	20	22
P13	12	13	14	15	12	14	16	19	12	13	16	18	13	15	17	20	-	-	-	-
P14	15	17	19	21	16	18	20	22	14	16	18	20	16	18	20	23	-	-	-	-
P15	15	17	18	20	14	15	16	18	12	13	14	16	-	-	-	-	-	-	-	-

A, B, C, D, E indicates concentration of compounds at 100 μg/ml, 200 μg/ml, 300 μg/ml, 400 μg/ml and 500 μg/ml, respectively. Ciprofloxacin (200 μg/ml) had 26 mm zone of inhibition for *S. aureus*, 25 mm for *B. subtilis*, 26 mm for *E. coli*, 30 mm for *P. aeruginosa* microorganisms respectively and fluconazole (100 μg/ml) had 28 mm zone of inhibition for *A. niger organism*. Figures indicate diameter of the zone of inhibition in mm including the diameter of cup (i.e. 10 mm). ‘-’ indicates resistance.
